# The role of miR-369-3p in proliferation and differentiation of preadipocytes in Aohan fine-wool sheep

**DOI:** 10.5194/aab-66-93-2023

**Published:** 2023-02-27

**Authors:** Shijie Xue, Kaidong Liu, Le Zhao, Lisheng Zhou, Xiaoxiao Gao, Lirong Liu, Nan Liu, Jianning He

**Affiliations:** 1 College of Animal Science and Technology, Qingdao Agricultural University, Qingdao 266109, China; 2 Qingdao animal husbandry and veterinary research Institute, Qingdao 266032, China; 3 China Animal Health and Epidemiology Center, Qingdao 266032, China

## Abstract

MicroRNAs (miRNAs) are a large class of non-coding RNAs that play important roles in the proliferation and differentiation of adipocytes. Our previous sequencing analysis revealed higher expression of miR-369-3p in the longissimus muscle of 2-month-old Aohan fine-wool sheep (AFWS) compared to 12-month-old sheep ( P<0.05 ), suggesting that miR-369-3p may regulate fat deposition in AFWS. To test this, miR-369-3p mimics, inhibitors, and negative controls (NCs) were constructed and transfected into AFWS preadipocytes. After transfection with miR-369-3p mimics, we found a decrease ( P<0.05 ) in the expression of genes and proteins related to cell proliferation and differentiation, detected by RT-qPCR (quantitative reverse transcription PCR) and western blot analyses. Moreover, EdU (5-ethynyl-2 ${}^{\prime }$ -deoxyuridine) detection and Oil Red O staining showed a decrease ( P<0.05 ) in cell proliferation and lipid accumulation, respectively. The opposite trends ( P<0.05 ) were obtained after transfection with miR-369-3p inhibitors. In conclusion, the results showed that miR-369-3p can inhibit the proliferation and differentiation of AFWS preadipocytes, providing a theoretical basis to further explore the molecular mechanism of fat deposition in sheep and other domestic animals.

## 1 Introduction

Mutton is becoming increasingly popular as living standards improve and dietary patterns change because of its unique taste, high protein content, and low cholesterol content (Jia et al., 2023). Intramuscular fat (IMF) content is a key indicator of meat quality, affecting meat colour, flavour, tenderness, and other traits, and increasing IMF relative to other indicators can improve the quality of meat (Mortimer et al., 2017). Therefore, determining how to improve IMF content is essential to improving the quality of meat.

MicroRNAs (miRNAs) are a class of 20–25 nt non-coding RNAs that regulate gene expression by targeting mRNA (Iorio et al., 2005; Yekta et al., 2004). Many miRNAs have been reported as regulators of fat formation and metabolism. For example, miR-145 downregulates *ADAM17* to promote the decomposition of human adipocytes (Lorente-Cebrián et al., 2014), miR-182 and miR-203 act in the differentiation of brown adipocytes (Kim et al., 2014), and miR-155 directly targets and inhibits *PPAR* γ expression to regulate adipocytes (Karkeni et al., 2016). Studies relating to sheep show that miR-27a regulated the formation of lipid droplets in sheep adipocytes by targeting the *CPT1B* gene (Li et al., 2021), and miR-340-5p inhibits sheep adipocyte differentiation by targeting *ATF7* (Liu et al., 2020). In a preliminary study, we evaluated the IMF content of 2-, 4-, 6-, and 12-month-old Aohan fine-wool sheep (AFWS). The results showed that the IMF content in the 2- and 12-month-old sheep was significantly different (Han et al., 2021a). Then we found many differentially expressed miRNAs in back longissimus muscle between 2-month- and 12-month-old AFWS by RNA-seq (Han et al., 2021b). Of these miRNAs, the expression level of miR-369-3p at 2 months old was significantly higher than at 12 months old (Table S1 in the Supplement). Therefore, miR-369-3p may have a regulatory effect on intramuscular fat content.

There have been few studies regarding the regulation of miR-369-3p on fat, whereas the majority of studies focused on disease. Galleggiante et al. (2019) showed that miR-369-3p inhibited the expression of *C/EBP* β . *C/EBP* β is the first set of transcription factors regulating the process of fat formation. Mouse embryonic fibroblasts (3T3-L1) lacking these genes cannot complete the differentiation of fat induced by hormones and interfere with normal expression of other adipogenic genes. *C/EBP* β also promotes cell mitosis in the process of fat formation. When induced in vitro, precursor adipocytes reenter the cell division cycle at the early stage of differentiation, and *C/EBP* β promotes cell mitosis in this process, which is required for adipocyte differentiation (Azad et al., 2014). The binding of miR-369-3p to the 3 ${}^{\prime }$ UTR region of *TSPAN13* mRNA inhibited expression of *TSPAN13*, leading to a decrease in *CDK4* expression (Li et al., 2019; Lou et al., 2017). *CDK4* indirectly inhibits oxidative metabolism by controlling *E2F1* transcription factors in muscle and brown adipose tissue (BAT) (Blanchet et al., 2011) and also promotes insulin signalling pathways in mature adipocytes (Lagarrigue et al., 2016). In sheep, miR-369-3p targets the key gene *PDPK-1* that activates autophagy, and its expression is negatively correlated (Lie et al., 2016; Hu et al., 2021). The miR-369-3p can also regulate and inhibit the autophagy-related gene *ATG10* (Liu et al., 2019).

Therefore, a significant amount of data has shown that miR-369-3p is closely related to genes related to adipocyte proliferation and differentiation. To further investigate the role of miR-369-3p in AFWS, AFWS preadipocytes were used to study the effect of miR-369-3p on preadipocyte proliferation and differentiation.

## 2 Materials and methods

### 2.1 Materials

Samples of preadipocytes, back longissimus muscle, biceps femoris, shoulder skin, pituitary, heart, lung, testicle, liver, and small intestine were collected from 4 d old Aohan fine-wool male lambs obtained from Aohan Breeding Farm in the city of Chifeng, Inner Mongolia. We anesthetized the sheep by intravenous injection of sodium pentobarbital at a dose of 25 mg kg ${}^{-1}$ following published protocols (Raj et al., 2004; Hawkins et al., 2016) and then placed it in a closed room that was filled with carbon dioxide at a rate of 20 % min ${}^{-1}$ . When the gas concentration had reached 80 %, the sheep died.

### 2.2 Differentially expressed miRNAs in sheep muscle tissue

In a preliminary study, we found many differentially expressed miRNAs in back longissimus muscle between 2-month- and 12-month-old AFWS by RNA-seq (Han et al., 2021b). Of these miRNAs, the expression level of miR-369-3p at 2 months old was significantly higher than at 12 months old (Table S1).

### 2.3 Culture and passage of AFWS preadipocytes

After the 4 d old AFWS was killed, the longissimus muscle tissue was taken and washed five times with PBS (phosphate-buffered saline)) containing 0.1 % penicillin / streptomycin. We also removed the visible blood vessels and connective tissue in sterile culture dishes. Then we cut the longissimus muscle tissue into a tissue block of about 1.0 mm, adding 0.2 % collagenase II (Solarbio, Beijing, China) before putting the tissue into a 37 ${}^{\circ }$ C water bath for digestion in 1.5 h. Shaking it every 5 min, we added a complete medium (Dulbecco's Modified Eagle Medium (DMEM) + 10 % fetal bovine serum (FBS) + 0.1 % penicillin / streptomycin) of equal volume after digestion. Digestive solution was filtered with 200 mesh and 400 mesh cell sieves, after which the cells were resuspended in the complete medium and placed in the CO ${}_{2}$ incubator for 3–4 h. The adherent cells were preadipocytes. When the confluence degree of primary cell culture reached 80 %, the cells were passaged at a ratio of 1 : 2 and placed in a 37 ${}^{\circ }$ C, 5 % CO ${}_{2}$ incubator.

### 2.4 Induction of preadipocyte differentiation

After 2 d of fusion, the cells were induced in the first stage after high cell fusion (DMEM, FBS, penicillin / streptomycin, methylisobutylxanthine, dexamethasone, insulin). Before induction, the cells were collected for RNA and protein extraction, which was recorded as day 0. After the first stage, some cells became round. After 2 d, the second stage of induction (DMEM, FBS, insulin, penicillin / streptomycin) was conducted. The culture medium was changed every 2 d, with cell collection.

### 2.5 Transfection

After the cells were digested using trypsin, they were evenly distributed in a six-well plate with 2 mL of complete medium and cultured overnight. When the density reached about 60 %, the mimics, inhibitors, and negative controls (NCs) (Jima Gene, Shanghai, China) were transfected into the cells with GP-transfect-Mate (Jima Gene, Shanghai, China) according to the manufacturer's instructions (A: miR-369-3p mimic, B: miR-369-3p mimic NC, C: miR-369-3p inhibitor, D: miR-369-3p NC). After 48 h, the cells were collected to detect the expression of miR-369-3p.

### 2.6 RNA extraction and reverse transcription

RNA was extracted from back longissimus muscle, biceps femoris, shoulder skin, pituitary, heart, lung, testis, spleen, and small intestine of 4 d old AFWS lambs. After grinding, total RNA was extracted using a SPARKeasy total RNA rapid extraction kit (Shandong Sikejie Biotechnology Co., Ltd.). The cDNA was prepared from the RNA using an Evo M-MLV reverse transcription kit (Aikerui Biotechnology, Hunan, China), followed by an miRNA cDNA first island chain synthesis kit (Aikerui Biotechnology, Hunan, China).

### 2.7 RT-qPCR (quantitative reverse transcription PCR)

The expression levels of mRNAs and miRNA were quantified by the CFX96 real-time PCR detection system (Bio-Rad, Hercules, CA, United States). Primers were designed using Primer 5 (Table S2) and synthesized by Tsingke Biological Technology (Tsingke, Qingdao, China). Reverse transcription was conducted using 1 µ g total RNA per sample and the Mir-X miRNA First-Strand Synthesis Kit (TaKaRa, Dalian, China). The levels of *U6* and *GAPDH* were used to normalize the expression levels of mRNA and miRNAs, respectively. The sequences of the primers used are listed in Table 1. The 20 µ L PCR reaction system consisted of 10 µ L SYBR^®^ Premix Ex Taq II (TaKaRa), 0.5 µ L forward primer (10 µ M L ${}^{-1}$ ), 0.5 µ L reverse primer (10 µ M L ${}^{-1}$ ), 1 µ L cDNA, and 8 µ L ddH ${}_{2}$ O. The following thermocycling programme was used: one cycle of 95 ${}^{\circ }$ C for 10 min; 45 cycles of 95 ${}^{\circ }$ C for 10 s, 60 ${}^{\circ }$ C for 10 s, and 72 ${}^{\circ }$ C for 10 s; and one cycle of 72 ${}^{\circ }$ C for 6 min. Three independent replicates were conducted for each sample. The relative expression levels were calculated using the 2 ${}^{-\Delta \Delta \mathrm{Ct}}$ method (Livak and Schmittgen, 2001).

### 2.8 Total protein extraction and western blot analysis

Total proteins were extracted using RIPA Lysis Buffer (Beyotime, Shanghai, China), supplemented with phenylmethylsulfonyl fluoride (Service-Bio, Wuhan, China) at a ratio of 100 : 1 on ice. The extracted protein concentration was then measured by a BCA Protein Assay Kit (Solarbio, Beijing, China) (Gao et al., 2019). Proteins (30 µ g) were separated by 10 % SDS-PAGE polyacrylamide gel electrophoresis and then transferred onto PVDF (polyvinylidene difluoride) membranes (Beyotime). The membranes were blocked with 5 % skimmed milk for 2 h and then incubated overnight at 4 ${}^{\circ }$ C with rabbit anti-*PPAR* γ , rabbit anti-*C/EBP* α , rabbit anti-*CDK4*, or rabbit anti-*CyclinB* at a dilution of 1 : 2000. The membranes were then incubated with a horseradish peroxidase (HRP)-conjugated secondary antibody (1 : 2000) for 1 h at room temperature, followed by washing. Rabbit anti-*GAPDH* was used as an internal control. All antibodies were purchased from Proteintech (Chicago, IL, United States). The protein signals were detected in a darkroom using a Pierce ECL Western Blotting Substrate kit (Thermo Fisher Scientific, Dallas, TX, United States) according to the manufacturer's instructions.

### 2.9 Oil Red O staining

Differentiation was induced in preadipocytes for 8 d, and then the cells were stained with Oil Red O (ORO; Solarbio, Beijing, China). The culture medium was removed, and cells were washed with PBS (phosphate buffered saline) and fixed with ORO fixative for 30 min. The fixative was removed, cells were rinsed with distilled water twice, and 60 % isopropanol was added and incubated for 5 min before removal. The ORO stain was then added and incubated for 15 min before removal. The stained cells were washed five times with distilled water. After washing, Mayer's hematoxylin staining solution was added, and cells were re-stained for 2 min. After discarding, the stained cells were washed five times with distilled water. ORO buffer was added, incubated for 1 min, and then discarded. Finally, the cells were covered with distilled water and observed under a microscope (Leica Microsystems, Wetzlar, Germany).

### 2.10 EdU detection of cell proliferation

After passage to the six-well plate, preadipocytes in good condition and at the growth density of 70 % were selected for EdU (5-ethynyl-2 ${}^{\prime }$ -deoxyuridine) staining using an EdU cell proliferation detection kit (Beyotime, Shanghai, China). Cells were fixed with 4 % paraformaldehyde and labelled with EdU, and then the nuclei were stained using Hoechst 33342.

### 2.11 Statistical analysis

IBM SPSS Statistics 26.0 software was used to calculate the relative expression level of genes using the 2 ${}^{-\Delta \Delta \mathrm{Ct}}$ method (Livak and Schmittgen, 2001). The relative expression of genes was analysed by an independent sample t test, and the results were expressed as mean ± SE (standard error). A value of P<0.05 was considered a significant difference.

## 3 Results

### 3.1 Expression of miR-369-3p in different AFWS tissues

**Figure 1:**
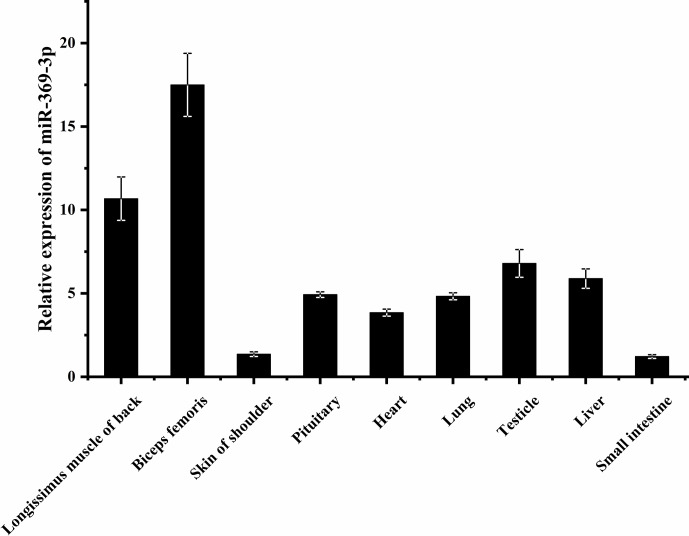
Expression of miR-369-3p in AFWS tissue.

To explore whether miR-369-3p mainly plays a role in sheep muscle, its expression on different AFWS tissues was determined. As shown in Fig. 1, RT-qPCR showed that miR-369-3p was widely expressed in various tissues, with higher expression levels in the back longissimus muscle and biceps femoris. As shown in Fig. S1 in the Supplement, comparison of the miR-369-3p sequences of 14 species revealed high conservation, indicating these miRNAs may play important roles.

### 3.2 The expression of miR-369-3p in preadipocyte differentiation

**Figure 2:**
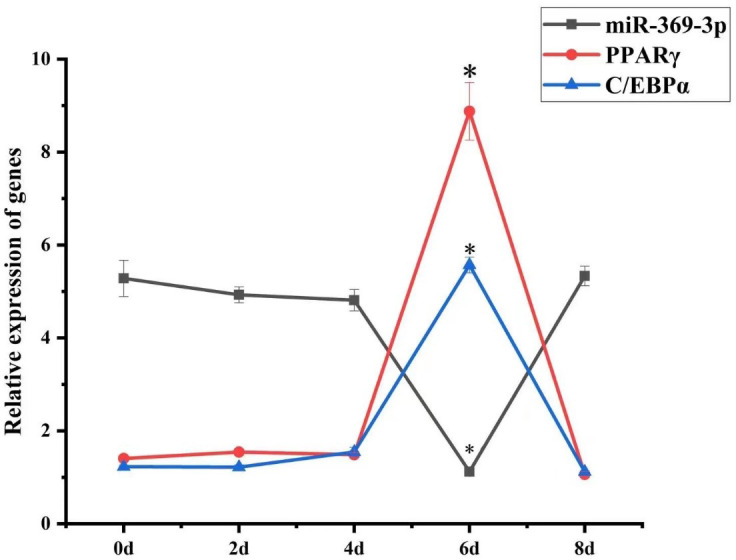
Temporal expression of miR-369-3p, *PPAR* γ , and *C/EBP* α in AFWS preadipocyte differentiation ( ${}^{*}$ P<0.05 ).

To explore the role of miR-369-3p in AFWS preadipocyte differentiation, its expression on consecutive days following induction of differentiation was determined. The results of RT-qPCR are shown in Fig. 2. The expression levels of marker genes for adipocyte differentiation, *PPAR* γ and *C/EBP* α , peaked at 6 d, and the differences in expression level were significant ( P<0.05 ). The expression levels decreased after 6 d, indicating a critical period for the differentiation of preadipocytes at 6 d, but the expression level of miR-369-3p reached the lowest value on the sixth day, with a significant difference.

### 3.3 Transfection effect of the miR-369-3p mimic and inhibitor

To further investigate the expression of miR-369-3p, we transfected cells with a mimic or inhibitor, as well as negative controls (NCs), as shown in Fig. S2. After 48 h of transfection, the expression of miR-369-3p in the mimic group was higher compared to the mimic NC group, and the difference was significant ( P<0.05 ). The expression of miR-369-3p in the inhibitor group was lower compared to the NC group, and this difference was significant ( P<0.05 ). The observed changes in expression indicated successful transfection of the miR-369-3p mimic and inhibitor, allowing for subsequent experiments.

### 3.4 The expression changes of preadipocyte proliferation and differentiation marker genes after transfection

**Figure 3:**
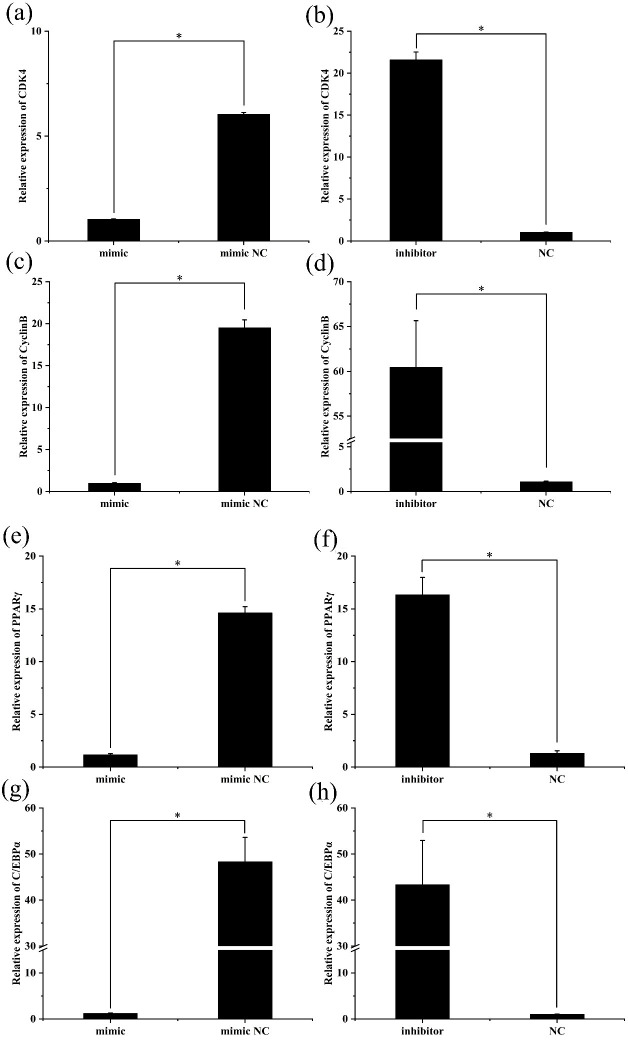
The expression levels of preadipocyte proliferation and differentiation marker genes after transfection. **(a)** Relative expression of *CDK4* after overexpression of miR-369-3p. **(b)** Relative expression of *CDK4* after inhibition of miR-369-3p. **(c)** Relative expression of *CyclinB* after overexpression of miR-369-3p. **(d)** Relative expression of *CyclinB* after inhibition of miR-369-3p. **(e)** Relative expression of *PPAR* γ after overexpression of miR-369-3p. **(f)** Relative expression of *PPAR* γ after inhibition of miR-369-3p. **(g)** Relative expression of *C/EBP* α after overexpression of miR-369-3p. **(h)** Relative expression of *C/EBP* α after inhibition of miR-369-3p ( ${}^{*}$ P<0.05 ).

To further investigate the role of miR-369-3p, the mimic or inhibitor constructs were used, and the effects on the expression levels of preadipocyte proliferation and differentiation marker genes were determined. As shown in Fig. 3, after transfection of the miR-369-3p mimic for 48 h, the expression levels of *CDK4*, *CyclinB*, and *PPAR* γ and of *C/EBP* α were significantly decreased ( P<0.05 ). However, the opposite results were obtained after transfection of the miR-369-3p inhibitor ( P<0.05 ).

### 3.5 Changes of protein expression after transfection

**Figure 4:**
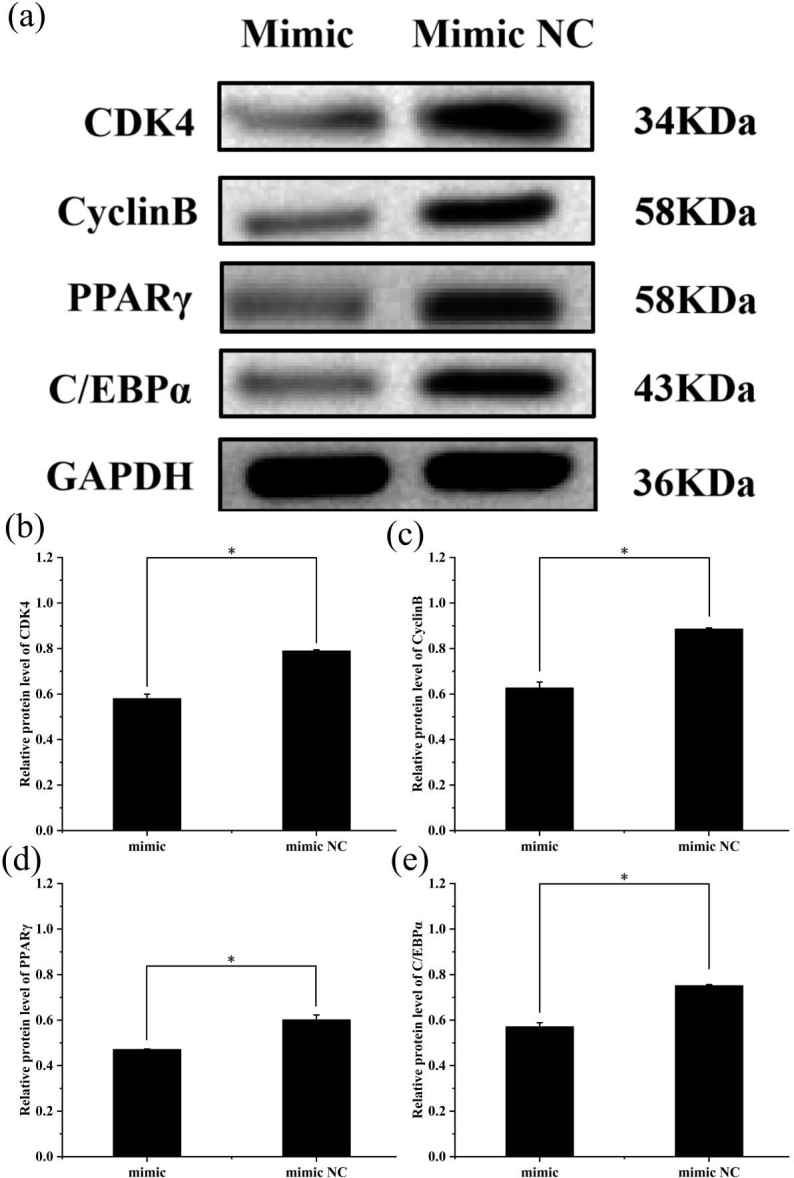
Effect of overexpression of miR-369-3p on the expression level of *CDK4*, *CyclinB*, *PPAR* γ , and *C/EBP* α . **(a)** Western blot analysis of adipogenic genes. **(b)** Relative protein level of *CDK4*. **(c)** Relative protein level of *CyclinB*. **(d)** Relative protein level of *PPAR* γ . **(e)** Relative protein level of *C/EBP* α ( ${}^{*}$ P<0.05 ).

**Figure 5:**
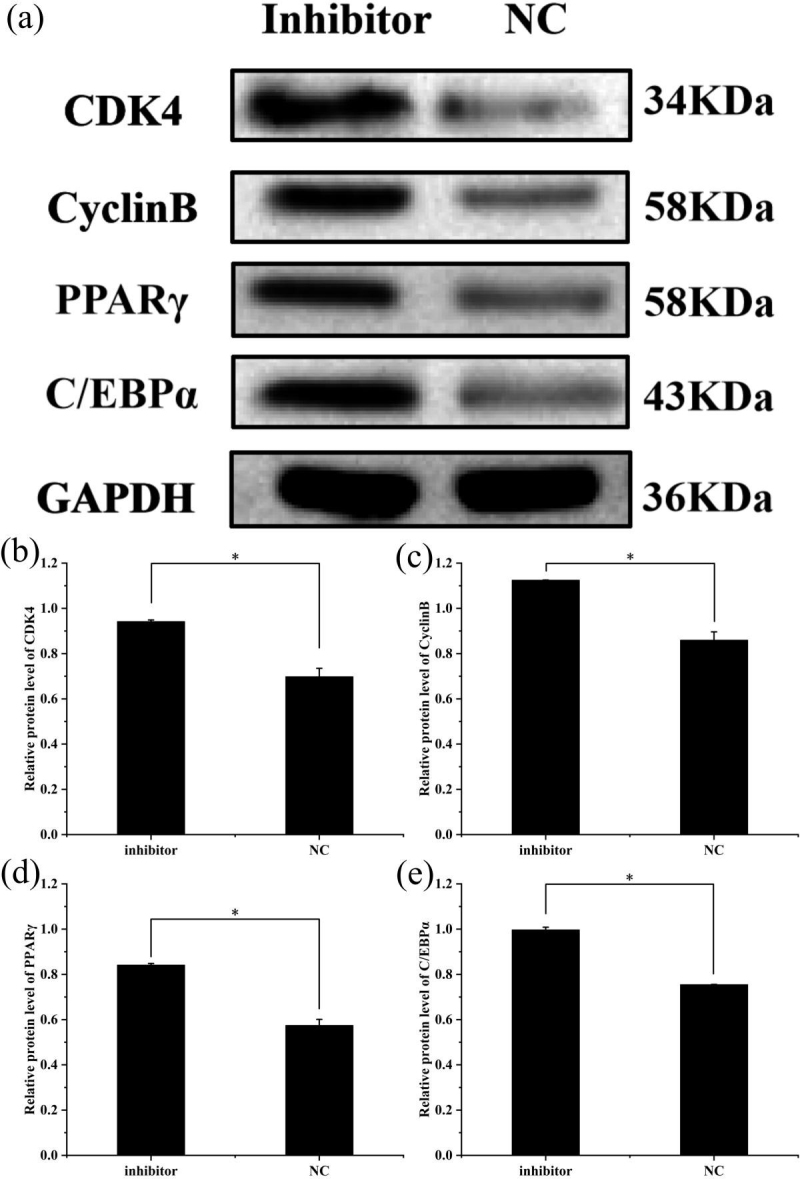
Effect of inhibition of miR-369-3p on the expression level of *CDK4*, *CyclinB*, *PPAR* γ , and *C/EBP* α . **(a)** Western blot analysis of adipogenic genes. **(b)** Relative protein level of *CDK4*. **(c)** Relative protein level of *CyclinB*. **(d)** Relative protein level of *PPAR* γ . **(e)** Relative protein level of *C/EBP* α ( ${}^{*}$ P<0.05 ).

To further explore the effect of miR-369-3p on the proliferation and differentiation of adipocytes, the total protein was extracted from preadipocytes after transfection for 48 h in each transfected group. The protein expression levels of adipocyte marker genes, *CDK4*, *CyclinB*, and *PPAR* γ , as well as *C/EBP* α , were determined, using the level of *GAPDH* as a control, as shown in Figs. 4 and 5. The *CDK4*, *CyclinB*, and *PPAR* γ as well as *C/EBP* α protein expression was inhibited by miR-369-3p mimics and promoted by miR-369-3p inhibitors. The results showed changes in adipocyte marker genes consistent with the observed trends in RNA expression detected by RT-qPCR.

### 3.6 EdU detection of cell proliferation

**Figure 6:**
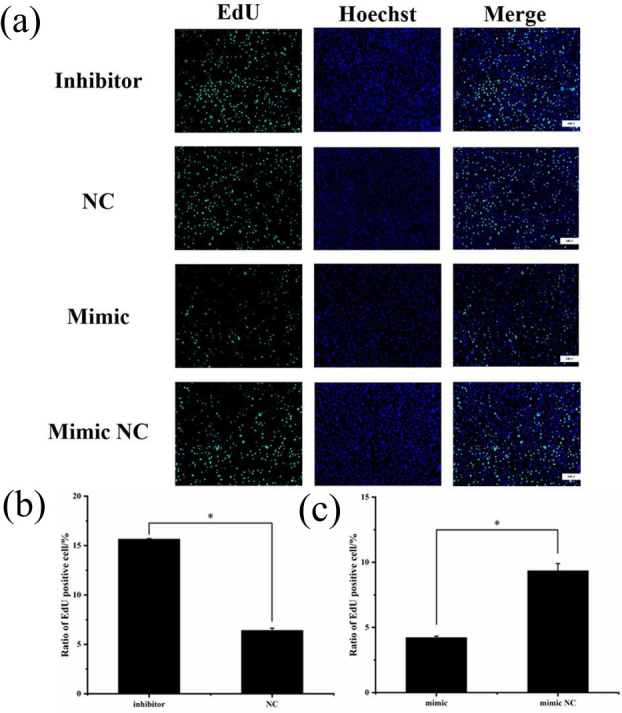
**(a)** EdU detection of cell proliferation after transfection. Blue fluorescence represents the nucleus stained with Hoechst, and green fluorescence represents proliferating cells. **(b)** Ratio of EdU-positive cell of inhibitor and NCs. **(c)** Ratio of EdU-positive cell of mimic and mimic NCs ( ${}^{*}$ P<0.05 ).

To assess cell proliferation, EdU detection was performed using the transfected cells, and the results are shown in Fig. 6. Cell proliferation was significantly greater in the miR-369-3p inhibition group compared to the NC group, and the opposite result was obtained in the mimic group ( P<0.05 ). Thus, miR-369-3p acts to inhibit the proliferation of preadipocytes.

### 3.7 Oil Red O staining

**Figure 7:**
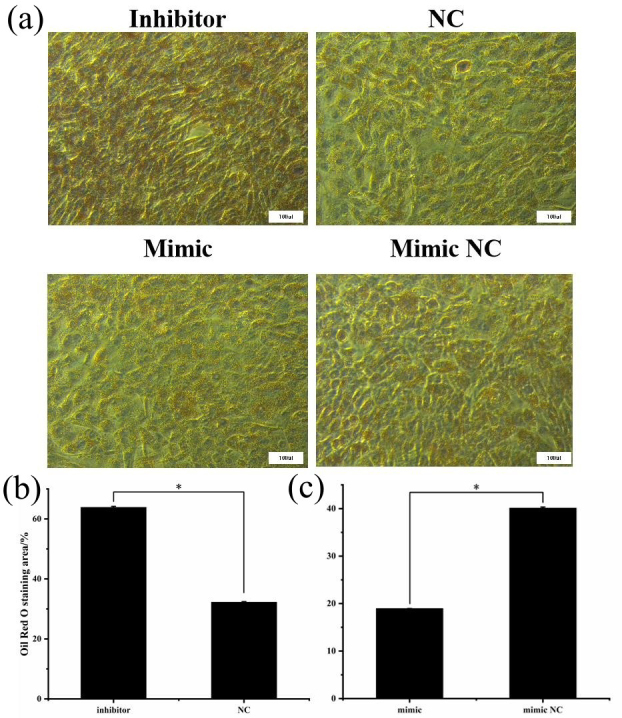
Oil Red O staining after transfection. **(a)** The red areas represent stained lipid droplets. **(b)** Oil Red O staining area of inhibitor and NCs. **(c)** Oil Red O staining area of mimic and mimic NCs ( ${}^{*}$ P<0.05 ).

After being induced for 8 d, the cells were stained with Oil Red O to investigate the effect of miR-369-3p on lipid deposition, as shown in Fig. 7. There was significantly greater staining in the miR-369-3p inhibitor group compared to the NC group, with the opposite results in the mimic group ( P<0.05 ). These results further verified that miR-369-3p can inhibit the differentiation of preadipocytes.

## 4 Discussion

As non-coding RNAs, miRNAs play important roles in a variety of tissues or organs by regulating gene expression. In adipose tissue, miRNAs can regulate adipocyte proliferation, differentiation, metabolism, and endocrine function (Arner and Kulyté, 2015). Our previous work revealed significantly lower expression of miR-369-3p in the longissimus dorsi muscle of 12-month-old AFWS compared to that of 2-month-old sheep (Han et al., 2021b) (Table S1). We found that the expression of miR-369-3p in muscle was higher compared to other tissues. This suggests that miR-369-3p acts in the growth and development of muscles. So far, studies on miR-369-3p have mainly focused on diseases, including cancer and inflammation (Chen et al., 2021; Scalavino et al., 2020; Xu and Liu, 2020). For example, in a study of chronic inflammation, Galleggiante et al. (2019) found that the upregulation of miR-369-3p could inhibit the expression of *C/EBP* β , a key transcription factor in the differentiation of preadipocytes (Cao et al., 2017). There was another study showing that during 3T3-L1 adipocyte differentiation, *C/EBP* β is induced early to transactivate the expression of *C/EBP* α and *PPAR* γ (Guo et al., 2015). Members of the *C/EBP* and *PPAR* families of transcription factors are the most notable in the differentiation of preadipocytes into adipocytes. Furthermore, *C/EBP* α and *PPAR* γ are critical regulators of adipogenesis, since the lack of either of these proteins prevents the development of white adipose tissue in mice (Linhart et al., 2001; Rosen et al., 1999; Barak et al., 1999). Therefore, miR-369-3p may inhibit the expression of *C/EBP* α and *PPAR* γ by inhibiting the expression of *C/EBP* β , thus inhibiting the differentiation of adipocytes.

Autophagy removes multiple cellular components during fat differentiation (Mizushima and Levine, 2010). Xu et al. (2018) showed that the expression of the autophagy pathway was negatively correlated with fat decomposition. *ATG10* plays an important role in the formation and extension of autophagosomes (Kim and Lee, 2014). Liu et al. (2019) found that miR-369-3p regulates the proliferation and migration of EEC (endometrioid adenocarcinoma) cells, suggesting that miR-369-3p can also inhibit adipocyte differentiation by inhibiting the expression of *ATG10* in human body. Lie et al. (2016) showed that miR-369-3p targeted *PDPK-1* (3-phosphoinositide-dependent protein kinase-1) in sheep, and expression levels were negatively correlated. *PDPK-1* is the key gene to initiate autophagy (Hu et al., 2021), and the expression of *PDPK-1* and *PPAR* γ resulted in a synergistic effect (Yin et al., 2006). Hence, miR-369-3p may inhibit the autophagy process and the expression of *PPAR* γ by inhibiting the expression of *PDPK-1* in sheep to negatively affect the differentiation of adipose precursor cells.

Lou et al. (2017) found that in CRC (colorectal cancer) cell lines, knocking down TSPAN13 led to the decrease of *CDK4* expression, and miR-369-3p inhibited *TSPAN13* by binding to the 3'UTR region of *TSPAN13* mRNA (Li et al., 2019). Therefore, miR-369-3p likely inhibits the expression of *CDK4* by downregulating *TSPAN13*. In previous studies, overexpression of *CDK4* has been shown to promote cell proliferation by driving cell cycle progression (Retzer-Lidl et al., 2007; Rodriguez-Puebla et al., 2002; An et al., 1999). Further, the proper regulation of *CyclinB* is essential for the entry into mitosis (Krek and Nigg, 1991), and reducing the time between cell birth and mitosis increases the rate of cell multiplication (Moxnes et al., 2004).

In summary, miR-369-3p negatively regulates proliferation and differentiation of AFWS preadipocytes.

## 5 Conclusion

In this study, we found that miR-369-3p inhibits the proliferation and differentiation of AFWS preadipocytes. This study provides a theoretical basis for further exploration of the molecular mechanism of fat deposition in sheep and other livestock.

## Supplement

10.5194/aab-66-93-2023-supplement The supplement related to this article is available online at: https://doi.org/10.5194/aab-66-93-2023-supplement.

## Data Availability

Additional data can be found in the Supplement.

## Appendix

The supplement related to this article is available online at: https://doi.org/10.5194/aab-66-93-2023-supplement.
