# Supplementary material for: The role of miR-369-3p in proliferation and differentiation of preadipocytes in Aohan fine-wool sheep
Source: Arch Anim Breed. 2023 Feb 27;66(1):93–102. doi: 10.5194/aab-66-93-2023 (PMC10294027; doi:10.5194/aab-66-93-2023)
Supplement: The supplement related to this article is available online at: https://doi.org/10.5194/aab-66-93-2023-supplement. [file aab-66-93-supplement.zip › The captions of the tables and figures.docx]

**Table S1** Summary of differentially expressed miRNAs.

**Table S2** Primer sequences.

Figure S1 The conservation of miR‐369-3p was determined by comparing the seed sequences of different species.Red represents adenine, yellow represents uracil, blue represents cytosine, and green represents guanine.Oar represents sheep, chi represents goat, cpo represents guinea pig, hsa represents human, eca represents horse, rno represents brown rats, mml represents rhesus monkey, ppy represents borneo orangutan, bta represents cattle, tch represents tree shrews, cja represents marmoset, mmu represents house mouse, dno represents armadillo, ocu represents rabbit, cgr represents Chinese hamster.

**Figure S2** Transfection effect of miR-369-3p mimic and inhibitor.(a)Relative expression of miR-369-3p after transfection of mimic and mimic NC.(b)Relative expression of miR-369-3p after transfection of inhibitor and NC.
