# Supplementary material for: The role of miR-369-3p in proliferation and differentiation of preadipocytes in Aohan fine-wool sheep
Source: Arch Anim Breed. 2023 Feb 27;66(1):93–102. doi: 10.5194/aab-66-93-2023 (PMC10294027; doi:10.5194/aab-66-93-2023)
Supplement: The supplement related to this article is available online at: https://doi.org/10.5194/aab-66-93-2023-supplement. [file aab-66-93-supplement.zip › aab-66-93-2023-supplement-title-page.pdf]

Supplement of Arch. Anim. Breed., 66, 93–102, 2023
https://doi.org/10.5194/aab-66-93-2023-supplement
© Author(s) 2023. CC BY 4.0 License.

Open Access
Archives Animal Breeding

Supplement of

The role of miR-369-3p in proliferation and differentiation of preadipocytes in Aohan fine-wool sheep

Shijie Xue et al.

Correspondence to: Nan Liu (nanliu@sina.com) and Jianning He (hexingxing104@163.com)

- aab-66-93-2023-supplement-title-page.pdf
- Figure S1 The conservation of miR-369-3p was determined by comparing the seed sequences of different species..png
- Figure S2 Transfection effect of miR-369-3p mimic and inhibitor..png
- Table S1 Summary of differentially expressed miRNAs.xlsx
- Table S2 Primer sequences.xls
- The captions of the tables and figures.docx

The copyright of individual parts of the supplement might differ from the article licence.
