# Supplementary figures and images for: The role of miR-369-3p in proliferation and differentiation of preadipocytes in Aohan fine-wool sheep
Source: Arch Anim Breed. 2023 Feb 27;66(1):93–102. doi: 10.5194/aab-66-93-2023 (PMC10294027; doi:10.5194/aab-66-93-2023)

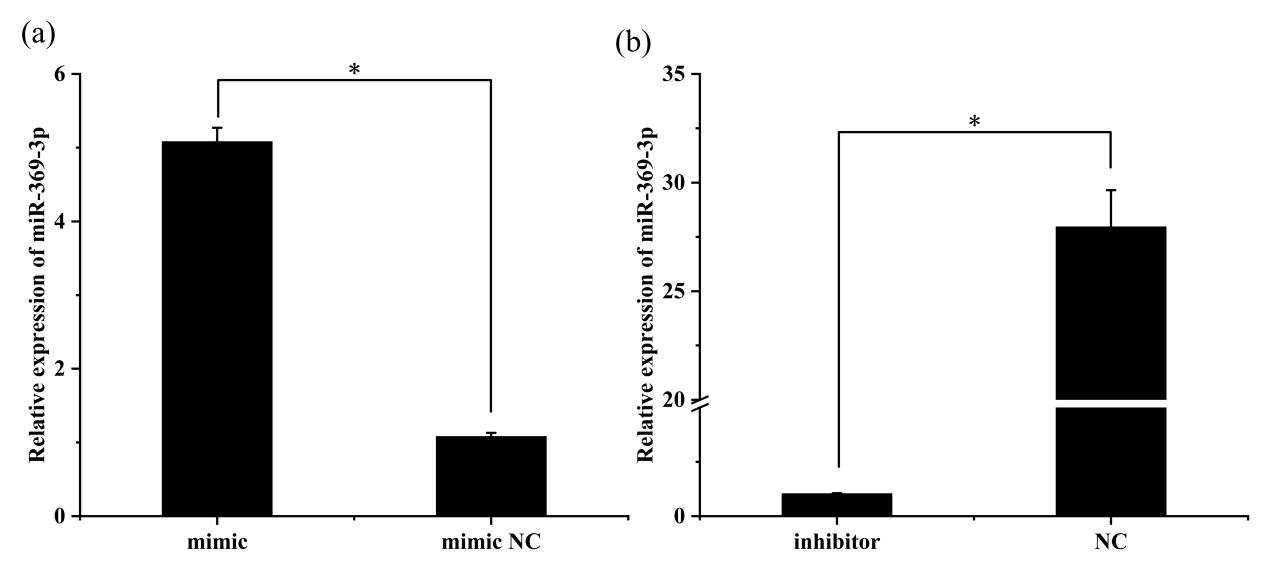

Supplement: The supplement related to this article is available online at: https://doi.org/10.5194/aab-66-93-2023-supplement. [file aab-66-93-supplement.zip › Figure S2 Transfection effect of miR-369-3p mimic and inhibitor..png]

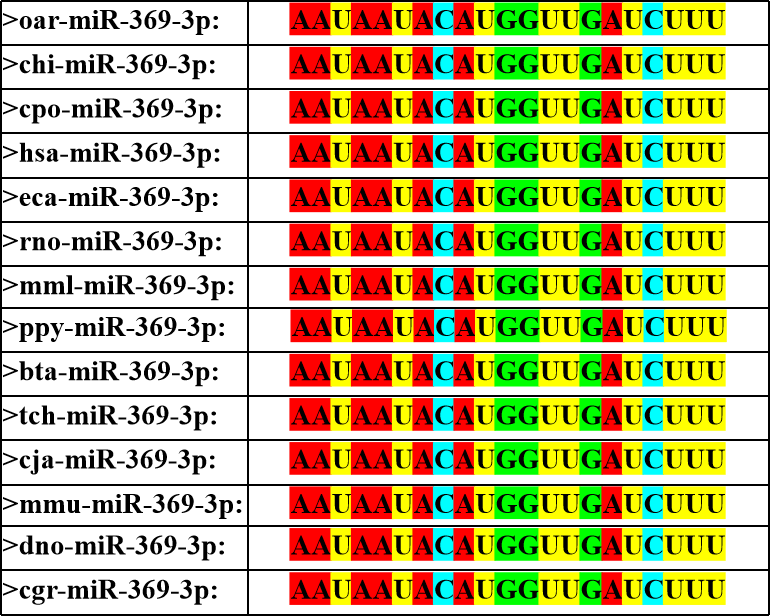

Supplement: The supplement related to this article is available online at: https://doi.org/10.5194/aab-66-93-2023-supplement. [file aab-66-93-supplement.zip › Figure S1 The conservation of miR‐369-3p was determined by comparing the seed sequences of different species..png]
